# Siteng Fang Reverses Multidrug Resistance in Gastric Cancer: A Network Pharmacology and Molecular Docking Study

**DOI:** 10.3389/fonc.2021.671382

**Published:** 2021-05-07

**Authors:** Lingjian Guo, Haixia Shi, Limin Zhu

**Affiliations:** ^1^ LongHua Hospital Shanghai University of Traditional Chinese Medicine, Shanghai, China; ^2^ Shanghai Ninth People’s Hospital Affiliated to School of Medicine, Shanghai Jiao Tong University, Shanghai, China

**Keywords:** Siteng Fang, gastric cancer, network pharmacology, molecular docking, multidrug resistance

## Abstract

Siteng Fang (STF) has been shown to inhibit migration, invasion, and adhesion as well as promote apoptosis in gastric cancer (GC) cells. However, whether it can reverse the multidrug resistance (MDR) of GC to chemotherapy drugs is unknown. Thus, we aimed to elucidate the mechanism of STF in reversing the MDR of GC. The chemical composition of STF and genes related to GC were obtained from the TCMNPAS(TCM Network Pharmacology Analysis System, TCMNPAS) Database, and the targets of the active ingredients were predicted using the Swiss Target Prediction Database. The obtained data were mapped to obtain the key active ingredients and core targets of STF in treating GC. The active component-target network and protein interaction network were constructed by Cytoscape and String database, and the key genes and core active ingredients were obtained. The biological functions and related signal pathways corresponding to the key targets were analyzed and then verified *via* molecular docking. A total of 14 core active ingredients of STF were screened, as well as 20 corresponding targets, which were mainly enriched in cancer pathway, proteoglycan synthesis, PI3K-AKT signaling pathway, and focal adhesion. Molecular docking showed that the core active ingredients related to MDR, namely quercetin and diosgenin, could bind well to the target. In summary, STF may reverse the MDR of GC and exert synergistic effect with chemotherapeutic drugs. It mediates MDR mainly through the action of quercetin and diosgenin on the PI3K/AKT signaling pathway. These findings are the first to demonstrate the molecular mechanism of STF in reversing MDR in GC, thus providing a direction for follow-up basic research.

## Introduction

Gastric cancer (GC) is one of five cancers with the highest incidence in China (1). According to the National Cancer Center, the incidence and mortality of GC ranked second among malignant tumors in China in 2015, and showed an increasing trend each year (2). Moreover, the early diagnosis rate of GC in China is low; patients with GC are often diagnosed with advanced disease. Thus, GC is a serious threat to the quality of life and health of the Chinese population. Chemotherapy is one of the important approaches to GC treatment. However, multidrug resistance (MDR) has become a common phenomenon, and it is a main reason for chemotherapy failure, making it difficult to improve the survival rate of patients with advanced GC (3). Therefore, overcoming the MDR of GC to chemotherapy and improving the efficacy of anticancer drugs are key issues in the global medical community.

Traditional Chinese medicine (TCM) has been used for the treatment of GC, and clinical studies have shown that TCM can improve the effect of western medicine, reduce the size and clinical stage of tumor, reverse MDR to chemotherapy drugs, relieve adverse reactions to chemotherapy, and improve the quality of life of patients (4). Weichang’an decoction was developed based on the clinical experience of Professor Qiu Jiaxin, a famous TCM doctor in Shanghai, China. It has been used in clinical setting as a hospital preparation for more than 30 years, and its efficacy has been confirmed by experimental studies. Weichang’an decoction can inhibit the metastasis and invasion of GC cells, induce apoptosis, regulate immunity, and regulate the expression of multiple genes (5); Siteng Fang (STF), which is composed of Radix Actinidiae Chinensis, wild grape, *Sargentodoxa* vine, and Chinaroot Greenbrier Rhizome Catbriar, is a small prescription of Weichang’an decoction. Its main efficacies are clearing away heat as well as detoxifying and eliminating pathogenic factors. Clinical research has demonstrated that STF improves the clinical efficacy of chemotherapy drugs and the quality of life of patients with GC (6–8). In addition, STF could inhibit the migration, invasion, and adhesion of GC cells and promote apoptosis (9). We have also previously observed a capacity of STF to reverse MDR (currently not reported). However the underlying molecular mechanism of MDR reversal remains unclear. Therefore, in this study, we aimed to clarify the molecular mechanism of STF in the treatment of GC. For this purpose, we used a combinatorial approach of network pharmacology and molecular docking technology. The findings of our study are the first to report the molecular mechanism of STF in reversing the MDR of GC, and thus will provide a direction for follow-up basic research.

## Materials and Methods

### Screening of Active Ingredients

The network pharmacology analysis system of TCM was developed by Yang Ming, the director of Longhua Hospital Affiliated to Shanghai University of traditional Chinese Medicine (TCM Network Pharmacology Analysis System, TCMNPAS, National Computer Software Registration No. 2019SR1127090) (10). The Chinese names of Radix *Actinidiae chinensis* (mihoutaogen), wild grape (yeputaogen), *Sargentodoxa* vine (daxueteng), and Chinaroot Greenbier Rhizome Catbriar (baqia) were input into the retrieval module of chemical constituents. The Traditional Chinese Medicine Systems Pharmacology Database and Analysis Platform (TCMSP) Version 2.3, Traditional Chinese Medicine Integrated Database (TCMID) Version 2.0, and the Herbal Ingredients’ Targets (HIT) Database were screened simultaneously, with TCMNPAS linked to the databases, to determine the chemical composition of STF (11–13). The active STF ingredients were screened based on oral bioavailability (OB) ≥ 30% and drug-likeness (DL) ≥ 0.18 (14) pharmacokinetic characteristics.

### Prediction of Drug Targets

To obtain the potential targets of the active ingredients of STF, the SMILES strings obtained from the TCMNPAS Database, were imported into the Swiss Target Prediction Database. Targets with 0 probability were deleted (15).

### Collection of GC Related Targets

Disease ID was obtained by inputting the disease keyword “gastric cancer” to the disease gene retrieval module of TCMNPAS. The background automatically connected to GeneCard Database to obtain disease gene and then downloaded and saved the results as GC related targets (16).

### Screening of Drug-Disease Key Targets

The targets of the active components of STF and GC-related targets were introduced into venny2.1.0 (https://bioinfogp.cnb.csic.es/tools/venny/index.html), and the intersection was regarded as the target protein of STF in treating GC. Cytoscape 3.7.0 (17) was used to analyze network topology parameters, and the active component-target-disease network diagram was constructed for visualization. Next, to obtain the protein interaction network diagram, the above-mentioned cross-proteins were input in String Database (https://string-db.org), the species was set as “Homo sapiens”, and the minimum interaction score was set as 0.4. Finally, the protein interaction data were imported into Cytoscape 3.7.0 to analyze the network topology parameters. The proteins above the median of “Degree” were selected as the key targets, and the corresponding chemical components were the core active components.

### Enrichment Analysis of Key Targets

The target obtained from section *Screening of Drug-Disease Key Targets* was transferred into DAVID6.8 (https://david.ncifcrf.Gov) for Gene Ontology (GO) functional enrichment analysis and Kyoto Gene and Genome Encyclopedia (KEGG) pathway enrichment analysis. The species was set as “Homo sapiens”, and the result was set as P<0.05. GO functional enrichment analysis describes the possible molecular functions of target products, the biological processes involved, and the cellular environment. KEGG pathway enrichment analysis indicates the most significant biological process by classifying the known genome annotation information. Therefore, these methods can predict the potential active components involved in the action mechanism of STF in treating GC.

### Molecular Docking Analysis

The protein IDs of the key targets were obtained from the utility module of TCMNPAS and converted into Protein Data Bank (PDB) IDs (every molecular model in the PDB has a unique accession or identification code). The 3D structure of the key proteins in PDB format were downloaded from the PDB Database (https://www.rcsb.org/). Subsequently, the SMILES strings of the active ingredient numbers, PDB IDs of the key targets, and 3D structure in PDB format obtained from 2.1 were inputted to the molecular docking module of TCMNPAS. The PSOVina algorithm was optimized based on the autodock Vina molecular docking algorithm and used PSOVina for docking (18–21). The energy range was set to 3, the accuracy was 8, and the output was 9 prediction results. The method of protein docking pocket parameters was FromLigand. Finally, the conformation and docking results of the compounds were downloaded and saved. The compound and target protein formats were converted into PDB format by Open Babel (22) and then imported into PyMOL 3.8 (https://pymol.org) to obtain 3D images of molecular docking. When the binding energy is less than -5 kJ/mol, the ligand is regarded to bind well to the receptor.

## Results

### Active Ingredients

In total, 112 components of STF were obtained from the TCMNPAS Database. After screening and removal of duplicate components according to the set Absorption, Distribution, Metabolism, and Excretion (ADME) parameters, 21 active components were obtained (**Table 1**).

**Table 1:** Active components list of Siteng Fang.

Number	Compound name	OB (%)	DL
1	beta-sitosterol	36.91	0.75
2	sitosterol	36.91	0.75
3	meso-1,4-Bis-(4-hydroxy-3-methoxyphenyl)-2,3-dimethylbutane	31.32	0.26
4	2-(4-hydroxyphenyl)ethyl (E)-3-(4-hydroxyphenyl)prop-2-enoate	93.36	0.21
5	(-)-catechin	49.68	0.24
6	saringosterol	43.48	0.62
7	methylprotodioscin_	35.12	0.86
8	pseudoprotodioscin_	37.93	0.87
9	Kaempferid	73.41	0.27
10	isoengelitin	34.65	0.7
11	Engelitin	36.27	0.7
12	(2R,3S)-2-(3,5-dihydroxyphenyl)chroman-3,5,7-triol	58.25	0.24
13	astilbin	36.46	0.74
14	taxifolin	57.84	0.27
15	maackoline	56.33	0.92
16	cis-Dihydroquercetin	66.44	0.27
17	diosgenin	80.88	0.81
18	aloe-emodin	83.38	0.24
19	(+)-catechin	54.83	0.24
20	ent-Epicatechin	48.96	0.24
21	quercetin	46.43	0.28

### Drug Targets

According to the prediction of Swiss Target Prediction Database, targets with 0 probability were removed; thus, six active components without target information were deleted. Finally, 709 potential targets of STF were obtained.

### Gastric Cancer-Related Targets

A total of 515 differentially expressed targets related to GC were identified. APC, CASP10, IRF1, MUTYH, erbB2, FGFR2, PIK3CA, KLF6, KRAS, and CTNNB1 were identified as the proteins with the highest scores.

### Drug-Disease Key Targets

**Figure 1:**
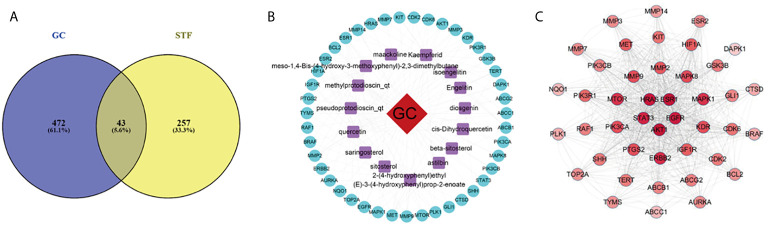
STF-GC gene mapping Venny map **(A)**; Active ingredients-target-disease network **(B)**; PPI network **(C)**.

From the intersection of the proteins obtained in sections *Drug Targets* and *Gastric Cancer-Related Targets*, 43 proteins involved in the mechanism of STF in treating GC were obtained (**Figure 1A**). The active component-target-disease network diagram was obtained by using Cytoscape 3.7.0. The blue round node represents the potential target of STF in treating GC, the purple rectangle node represents the active component of STF, and the red diamond node represents the targets of GC (**Figure 1B**). The protein interaction was predicted by using the String Database and visualized by Cytoscape 3.7.0. The darker the node, the closer the protein interaction. The top five proteins were AKT1, ESR1, HRAS, EGFR, and STAT3 (**Figure 1C**). According to analysis of network topology parameters, the proteins with more than median Degree of node were selected as key targets. The network contained a total of 20 target proteins (**Table 2**) and 14 core active components (**Table 3**).

**Table 2:** Key targets of STF decoction in the treatment of GC.

Number	Protein Name	Degree
1	AKT1	38
2	ESR1	37
3	HRAS	35
4	EGFR	34
5	STAT3	32
6	ERBB2	30
7	MTOR	29
8	MAPK1	29
9	MAPK8	25
10	PI3KCA	24
11	MMP9	24
12	PTGS2	24
13	KDR	24
14	MMP2	22
15	IGF1R	21
16	HIF1A	21
17	MET	21
18	PIK3R1	19
19	KIT	17
20	CDK6	17

**Table 3:** Core components of STF in the treatment of GC.

Number	Active Components	Degree
1	Kaempferid	21
2	quercetin	20
3	2-(4-hydroxyphenyl)ethyl (E)-3-(4-hydroxyphenyl)prop-2-enoate	18
4	meso-1,4-Bis-(4-hydroxy-3-methoxyphenyl)-2,3-dimethylbutane	11
5	isoengelitin	6
6	methylprotodioscin_qt	5
7	astilbin	5
8	Engelitin	4
9	diosgenin	4
10	sitosterol	3
11	saringosterol	3
12	beta-sitosterol	2
13	pseudoprotodioscin_qt	2
14	maackoline	2

### Enrichment Analysis of Key Targets

**Figure 2:**
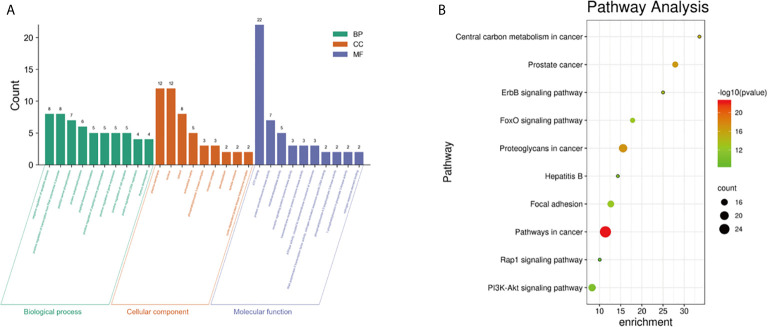
Top 10 GO enrichment classification histogram **(A)**; KEGG enrichment bubble diagram **(B)**.

A total of 41 selected key targets were analyzed for GO functional and KEGG pathway enrichment analyses and screened at P<0.05. A total of 52 GO biological processes, 9 cell components, 13 molecular functions (**Figure 2A**), and 86 KEGG pathways (**Figure 2B**) were enriched. The enriched molecular functions were ATP binding, protein serine/threonine kinase activity, and metalloendopeptidase activity. The biological processes were mainly involved in the negative regulation of apoptosis, positive regulation of RNA polymerase II promoter transcription, and phosphorylation of peptide serine. The cell components were the cell membrane, nucleus, and extracellular matrix. The KEGG pathways were the pathway in cancer, proteoglycan synthesis, PI3K/AKT signaling pathway, focal adhesion, and FOXO signaling pathway.

### Molecular Docking Analysis

**Figure 3:**
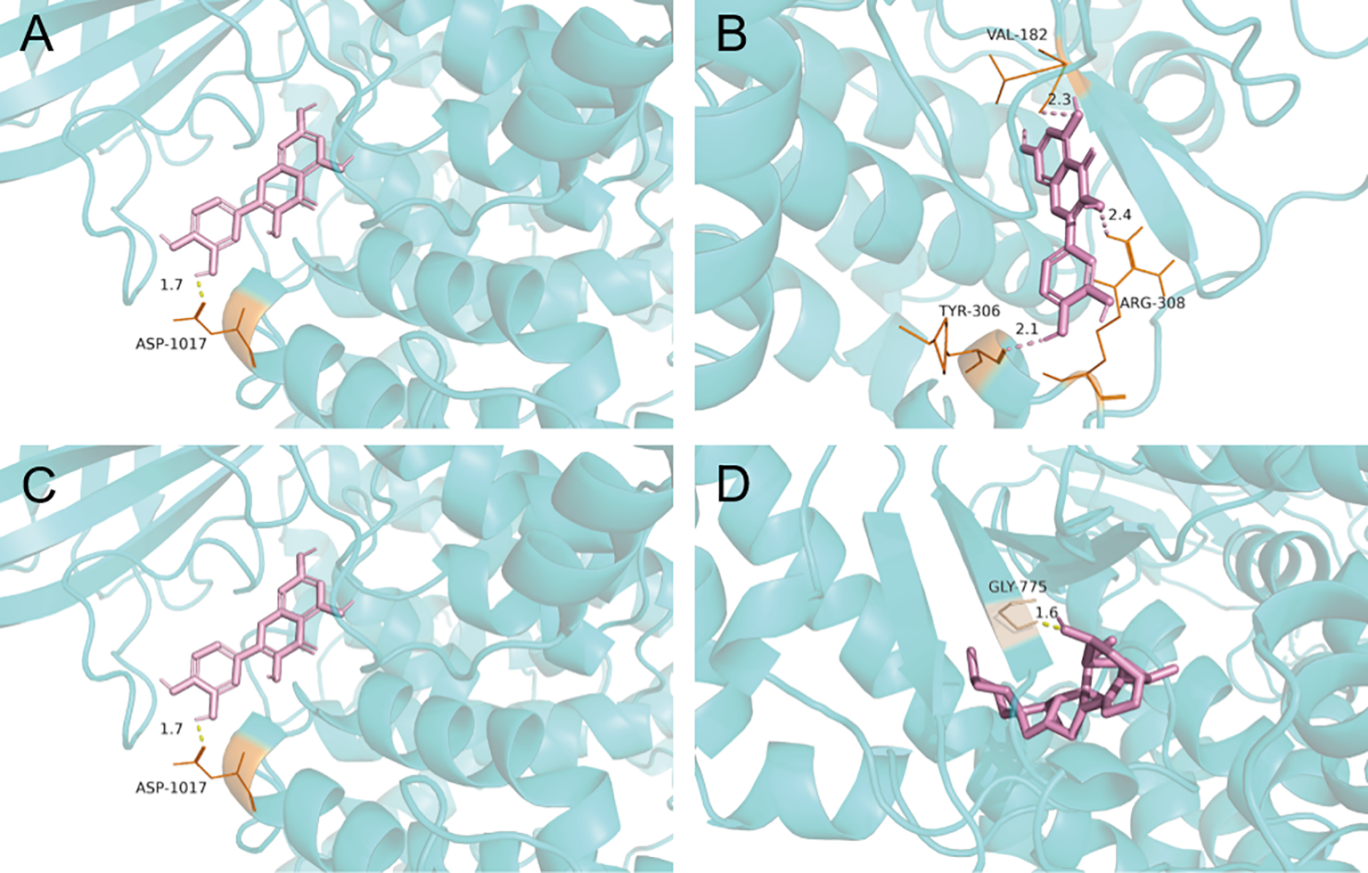
Molecular docking pattern map of quercetin-PI3K **(A)**; Molecular docking pattern map of quercetin-AKT **(B)**; Molecular docking pattern map of diosgenin-PI3K **(C)**; Molecular docking pattern map of diosgenin-AKT **(D)**.

Two of the screened active components, quercetin and diosgenin, were potentially associated with MDR, as discussed below, and the PI3K/AKT pathway was screened as one of the top 10 KEGG pathways related to MDR. These active components were subjected to molecular docking to the receptor proteins of the PI3K/AKT pathway. The results showed that the binding energies of quercetin and diosgenin with the target receptor protein were less than -5 kJ/mol, indicating that they could bind well to the target receptor (**Table 4**). The molecular docking diagram is shown in **Figure 3**. The results confirmed that quercetin and diosgenin could interact with PI3K and AKT. Quercetin and PI3K formed a hydrogen bond through ASP1017. Quercetin and AKT formed three hydrogen bonds through VAL182, TYR306, and ARG308. Diosgenin and PI3K formed a hydrogen bond through ASP1017. Diosgenin and AKT formed a hydrogen bond through GLY775. Quercetin and AKT formed the highest number of hydrogen bonds, and all the binding sites of quercetin and diosgenin with PI3K were on the ASP1017 residue, indicating that quercetin and diosgenin were the two core active components of STF and that the effect of STF in treating GC and reversing MDR might be mediated by the PI3K/AKT signaling pathway.

**Table 4:** The binding energy of key molecules and core targets.

Core active component	Binding energy (KJ/mol)	
	PI3K	AKT
quercetin	-9.0	-8.1
diosgenin	-6.9	-7.9

## Discussion

The aim of this study was to elucidate the molecular mechanism of STF in treating GC. A total of 14 core active components of STF were screened out, including kaempferol, quercetin, isoengelitin, methylprotodioscin, and diosgenin. The key signaling pathways included the PI3K/AKT signaling pathway, FOXO signal pathway, and ERBB signaling pathway. Basic studies have confirmed that quercetin, diosgenin, and the PI3K/AKT signaling pathway can regulate MDR to chemotherapeutic drugs. Quercetin is one of the active components of Radix *Actinidiae Chinensis*, and studies had shown that in addition to anti-inflammatory, antioxidant, apoptosis-inducing, and antiangiogenesis effects, quercetin has chemosensitizing effect to enhance the sensitivity of drug-resistant cells to drugs. The effects of quercetin on GC cells have been studied by Sylwia Borska et al. (23) using EPG85-257P cells and daunorubicin-resistant EPG85-257RDB cells. The results showed that quercetin inhibited the growth of the sensitive EPG85-257P cells and had a synergistic effect with daunorubicin. In the drug-resistant EPG85-257RDB cells, quercetin acted as a chemosensitizer, and the drug resistance mechanism of these cells might be related to a decrease in p-glycoprotein (p-gp) expression, obstruction of drug transport, and downregulation of ABCB1 gene expression. The study suggested that quercetin may be effective in reversing the classical drug resistance of GC cells. Zhaolin Chen et al. (24) confirmed that quercetin can increase the accumulation of rhodamine 123 and adriamycin, increase the sensitivity of BEL/5-FU cells to chemotherapeutic drugs, and downregulate the expression of ABCB1, ABCC1, and ABCC2, and that its effect was dependent on FZD7 through the Wnt/β-catenin pathway; quercetin can, at least partially, reverse chemotherapy resistance by inhibiting FZD7. Thus, this compound can be developed into an effective natural sensitizer to reverse the drug resistance of human liver cancer. Diosgenin is one of the active components of Chinaroot Greenbier Rhizome Catbriar, which has pharmacological effects, including anti-inflammatory, anticancer, antiviral, and hypotensive effects. Bu Tong Sun et al. (25) screened candidate MDR inhibitors among more than 300 natural compounds and revealed that diosgenin exerted inhibitory effect on MDR1 promoter activity. Experiments showed that diosgenin decreased the MDR of HepG2/adriamycin cells, significantly inhibited the expression of P-gp, and increased the accumulation of adriamycin in HepG2/adriamycin cells, indicating that diosgenin is an effective MDR reversal agent and a potential adjuvant drug for tumor chemotherapy. Another study suggested that diosgenin can reverse MDR to adriamycin by inhibiting the nuclear factor kappa B (NF-κB) signaling pathway and downregulating MDR1 expression (26). The PI3K/AKT signaling pathway is the main driving force of various cell functions. Excessive activation of this pathway plays a key role in cancer progression; it can promote tumorigenesis by regulating nutrient metabolism, cell proliferation, cell migration, and angiogenesis. Moreover, abnormal activation of PI3K/AKT is key to the regulation of MDR, mainly through the expression of death-related protein, ABC transporter, and glycogen synthase kinase-3β (GSK-3β), as well as synergistic effect with NF-κB and mammalian target rapamycin (mTOR). Some studies have suggested that P-gp and BCRP can be downregulated by PI3K110α and -110β to restore the drug sensitivity of drug-resistant human epidermoid carcinoma and non-small cell lung cancer, and that ABC family proteins and AKT may play an independent role in enhancing MDR (27–29).

In addition, preliminary research on the mechanism of STF in treating GC showed that STF can inhibit the growth of GC MGC-803 cells by regulating the Smac/Survivin signaling pathway *in vitro*, reduce the adhesion and invasion ability of SGC-7901 cells, inhibit cell migration, and induce apoptosis. Moreover, it can inhibit the growth of SGC-7901 GC cell xenograft tumor in nude mice *in vivo*; this effect may be related to the promotion of apoptosis, upregulation of the expression of apoptosis-related proteins caspase-8 and caspase-9, promotion of PARP editing, and downregulation of Livin protein expression (30–34). STF was also shown to inhibit the invasion, migration, and adhesion of GC cells and promote apoptosis, as confirmed by previous research. Furthermore, at the molecular level, molecular docking analysis showed that quercetin and diosgenin bound well with the active sites of PI3K and AKT, the key targets of the PI3K/AKT pathway, which verified the accuracy of this study to some extent.

In conclusion, this study preliminarily explored the potential molecular mechanism of STF in reversing the MDR of GC to chemotherapy through network pharmacology and molecular docking technology. The present study revealed quercetin and diosgenin as the core active components of STF as well as the PI3K/AKT signaling pathway as an important pathway involved in the effect STF in reversing the MDR of GC. Taken together, our results suggested that STF has the effect of reversing the MDR of GC and that it can play a synergistic effect with chemotherapy drugs. STF mainly reversed MDR through the action of quercetin and diosgenin on the PI3K/AKT signaling pathway. These findings provide a direction for future studies to further explore the mechanism of STF in treating GC. However, these conclusions were made based on theoretical simulations and thus still need to be verified by experiments. The authors’ team will conduct experiments as a follow-up to the present work.

## Data Availability Statement

All data generated or analyzed during this study are included in this article.

## Author Contributions

LG conceptualized and designed the study. LG acquired, analyzed and interpreted the data. LG and HS drafted/revised the work for intellectual content and context. LZ gave the final approval and overall responsibility for the published work. All authors contributed to the article and approved the submitted version.

## Funding

The research was funded by Shanghai Science and Technology Commission Development Foundation, award number is 16ZR1437500; Shanghai Health and Family Planning Commission of Medical Science and Technology Innovation Project, award number is ZYKC201701009.

## Conflict of Interest

The authors declare that the research was conducted in the absence of any commercial or financial relationships that could be construed as a potential conflict of interest.

The reviewer QX declared a shared affiliation, with no collaboration, with one of the authors HS to the handling editor at the time of the review.
